# IL-33-Dependent Endothelial Activation Contributes to Apoptosis and Renal Injury in *Orientia tsutsugamushi*-Infected Mice

**DOI:** 10.1371/journal.pntd.0004467

**Published:** 2016-03-04

**Authors:** Thomas R. Shelite, Yuejin Liang, Hui Wang, Nicole L. Mendell, Brandon J. Trent, Jiaren Sun, Bin Gong, Guang Xu, Haitao Hu, Donald H. Bouyer, Lynn Soong

**Affiliations:** 1 Department of Microbiology and Immunology, University of Texas Medical Branch, Galveston, Texas, United States of America; 2 Department of Pathology, Center for Biodefense and Emerging Infectious Diseases, Center for Tropical Diseases, Sealy Center for Vaccine Development, Institute of Human Infections and Immunity, University of Texas Medical Branch, Galveston, Texas, United States of America; University of Tennessee, UNITED STATES

## Abstract

Endothelial cells (EC) are the main target for *Orientia tsutsugamushi* infection and EC dysfunction is a hallmark of severe scrub typhus in patients. However, the molecular basis of EC dysfunction and its impact on infection outcome are poorly understood. We found that C57BL/6 mice that received a lethal dose of *O*. *tsutsugamushi* Karp strain had a significant increase in the expression of IL-33 and its receptor ST2L in the kidneys and liver, but a rapid reduction of IL-33 in the lungs. We also found exacerbated EC stress and activation in the kidneys of infected mice, as evidenced by elevated angiopoietin (Ang) 2/Ang1 ratio, increased endothelin 1 (ET-1) and endothelial nitric oxide synthase (eNOS) expression. Such responses were significantly attenuated in the IL-33^-/-^ mice. Importantly, IL-33^-/-^ mice also had markedly attenuated disease due to reduced EC stress and cellular apoptosis. To confirm the biological role of IL-33, we challenged wild-type (WT) mice with a sub-lethal dose of *O*. *tsutsugamushi* and gave mice recombinant IL-33 (rIL-33) every 2 days for 10 days. Exogenous IL-33 significantly increased disease severity and lethality, which correlated with increased EC stress and activation, increased CXCL1 and CXCL2 chemokines, but decreased anti-apoptotic gene BCL-2 in the kidneys. To further examine the role of EC stress, we infected human umbilical vein endothelial cells (HUVEC) *in vitro*. We found an infection dose-dependent increase in the expression of IL-33, ST2L soluble ST2 (sST2), and the Ang2/Ang1 ratio at 24 and 48 hours post-infection. This study indicates a pathogenic role of alarmin IL-33 in a murine model of scrub typhus and highlights infection-triggered EC damage and IL-33-mediated pathological changes during the course of *Orientia* infection.

## Author Summary

Scrub typhus is a life-threatening disease, caused by infection with *O*. *tsutsugamushi*, a Gram-negative bacterium that preferentially infects and replicates in the endothelium. Every year, approximately one million people are infected globally, especially in the Asia-Pacific region. However, the molecular mechanism(s) of tissue pathogenesis and immune responses in scrub typhus remain poorly understood. IL-33 is a damage-associated molecular pattern factor, which can modulate host inflammatory responses in several infectious diseases. In this study, we compared the severity of disease between wild-type (WT) and IL-33^-/-^ mice infected with *O*. *tsutsugamushi* and used exogenous IL-33 to further examine the function of IL-33 during the infection. Our studies in mouse models, as well as *in vitro* studies in human endothelial cells, have revealed a pathogenic role of IL-33 in promoting endothelial cell stress, cellular apoptosis, tissue damage, and host death. This study will help us understand the pathogenesis of severe scrub typhus.

## Introduction

*Orientia tsutsugamushi* is an obligately intracellular bacterium and the etiological agent of scrub typhus with a geographical distribution that encompasses much of the Asia-Pacific region [1]. Scrub typhus is a neglected but important tropical disease, which puts one-third of the world’s population at risk. The disease is transmitted by the bite of an infected larval *Leptotrombidium* mite or chigger. After 7–14 days of incubation, patients exhibit signs of infection such as an inoculation site eschar followed by fever and rash accompanied by non-specific flu-like symptoms. Although the endothelial tropism of *Orientia* can lead to disseminated endothelial infection that affects all organs; macrophages, dendritic cells and cardiac myocytes are also the targets of infection [2, 3]. Primary characteristics of fatal scrub typhus pathology include diffuse interstitial pneumonia, hepatic lesions, glomerulonephritis, meningoencephalitis, and coagulation disorders [3–6]. Scrub typhus often presents as an acute febrile illness [1, 7]. Without appropriate treatment, scrub typhus can cause severe multi-organ failure with a relatively high mortality rate [8]. Several antibiotics (doxycycline, azithromycin, rifampicin, chloramphenicol, etc.) have been used to treat *Orientia* infection. Although these antibiotics are effective if given early [9–12], misdiagnosis, inappropriate antibiotic treatment, and antibiotic failures have occurred, emphasizing the need for a vaccine and alternative therapeutics [1]. Understanding the molecular mechanism of the infection will be beneficial for vaccine design and future therapeutic strategies.

Infection-induced renal dysfunction, as well as acute kidney injury, has often been described in moderate-to-severe scrub typhus [13–18]. The severity of disease is often correlated with the extent of renal dysfunction [13, 17, 19, 20]; however, the molecular mechanism that accounts for such renal dysfunction is poorly understood. The endothelium provides a crucial interface between tissues and circulating inflammatory cells. During tissue damage, endothelial cells (ECs) become activated, expressing adhesion molecules that alert circulating leukocytes to possible insults and further allow the leukocyte to transmigrate across the endothelial layer. In the case of scrub typhus, ECs are the primary target cells once the bacteria has disseminated [3, 21]. During infection the ECs become activated, attracting inflammatory cells, resulting in the observed pathology. Endothelial activation and dysregulation can lead to tissue damage and organ dysfunction. Understanding the molecules that are released during infection is crucial to understanding the role of ECs in the host response during scrub typhus.

Damage-associated molecular pattern molecules (DAMPs) are molecules that can initiate and perpetuate an immune response within the noninfectious and infectious inflammatory responses. Among them, IL-33, a member of interleukin-1 family, locates in the nucleus as a chromatin-associated nuclear factor. IL-33 can modulate inflammatory responses when released [22, 23]. In damaged tissues, necrotic cells can directly release endogenous IL-33, which can signal through its receptor IL-33R/ST2L on target cells [24, 25]. IL-33 has pro- or anti-inflammatory roles, depending on the disease models and tissues involved [26]. For example, recombinant IL-33 (rIL-33) treatment can exacerbate cisplatin-induced acute kidney injury by increasing CD4^+^ T cell infiltration, CXCL1 production, and acute tubular necrosis [27]. IL-33 also mediates inflammatory responses in human lung tissue cells involved in the chronic allergic inflammation of the asthmatic airway [28]. However, IL-33 can be hepatoprotective in viral infection and ischemia/reperfusion-induced acute liver injury [29, 30]. At present, the role of IL-33 in severe scrub typhus is unclear.

In this study, we found that mice infected with *O*. *tsutsugamushi* Karp strain had significantly increased expression levels of IL-33 and its receptor in the kidneys and liver, but not in the lungs. IL-33 deficiency resulted in decreased kidney cellular infiltration and apoptotic cells, as well as delayed bodyweight loss. Compared to WT mice, the endothelium stress and activation in the kidneys of IL-33^-/-^ mice were significantly attenuated, as evidenced by increased angiopoietin (Ang) 1 and endothelial nitric oxide synthase (eNOS), and decreased endothelin-1 (ET-1). To further confirm the role of IL-33 in scrub typhus, we injected rIL-33 to sub-lethally infected mice, and observed the exacerbated illness and increased mortality. Moreover, rIL-33 treatment resulted in increased vascular dysregulation in the kidneys of infected mice. *In vitro*, *Orientia* infection significantly stimulated IL-33 and ST2 expression in human EC and increased EC activation. These data suggest that IL-33 plays a significant role in modulating host immune and endothelial responses during scrub typhus infection.

## Results

### Differential gene expression of IL-33 and ST2L during *Orientia* infection

**Fig 1:**
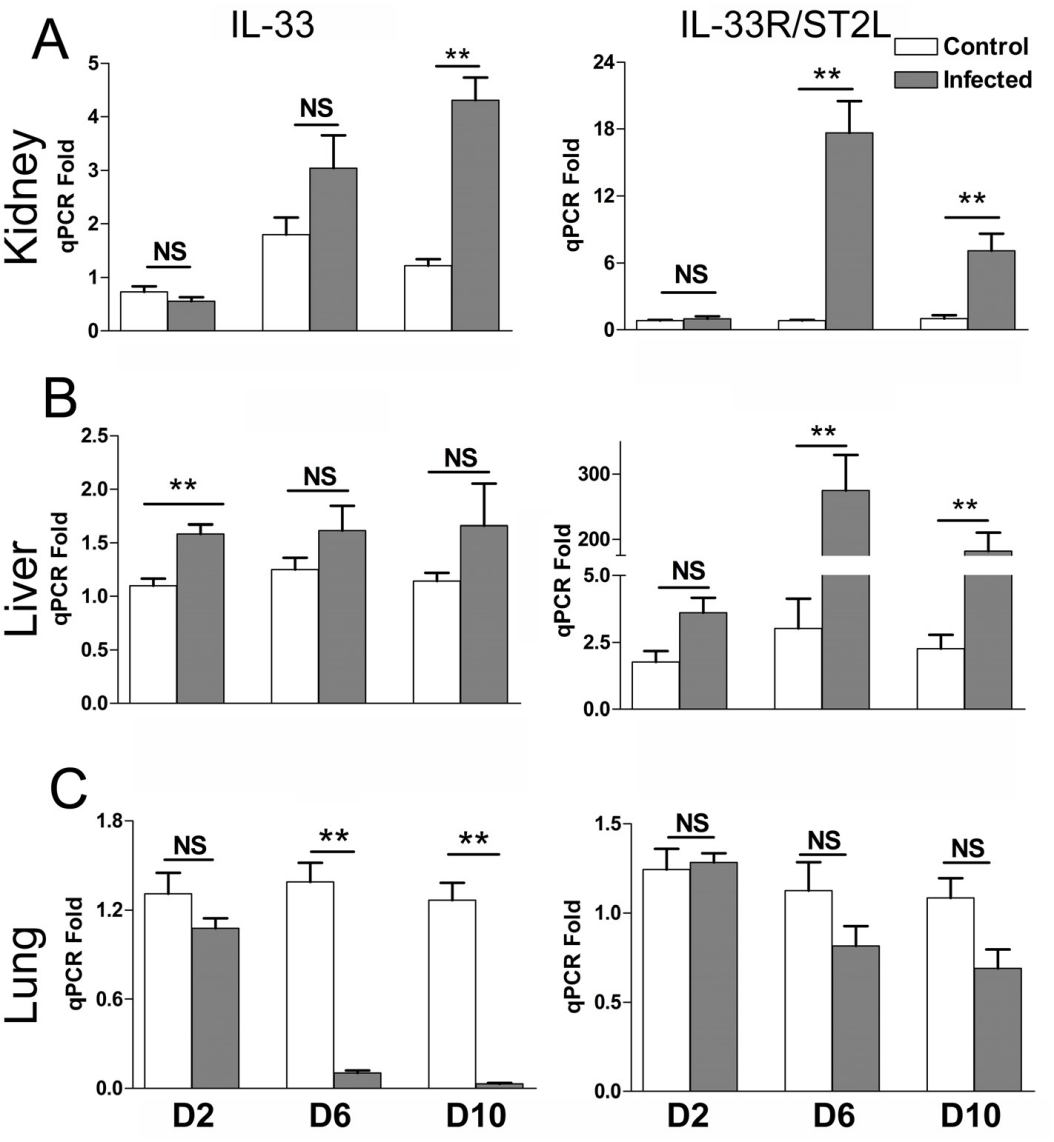
Expression of the IL-33 and IL-33R/ST2L genes during *Orientia* infection. C57BL/6J mice (4-5/group) were inoculated *i*.*v*. with *O*. *tsutsugamushi* Karp strain (4.5 x 10^6^ FFU, gray bars) or with PBS (open bars). At 0, 2, 6 and 10 days post-infection (dpi), total RNA was extracted from indicated tissues, and mRNA levels of IL-33 and ST2L in the kidneys (A), livers (B) and lungs (C) were analyzed by qRT-PCR. Data are presented as “qPCR fold” (after normalization to the house-keeping genes), and are shown as mean ± SEM in each group. Representative results are shown from two independent studies with similar trends. **, *p* < 0.01; NS, no significance.

We have recently reported a strong type 1 immune response, but a repressed type 2 response, accompanied with severe tissue damage in multiple organs during lethal infection with *O*. *tsutsugamushi* Karp strain in B6 mice [31, 32]. Since *Orientia* lacks the classical ligands for TLR2/4 stimulation, we speculated that the host DAMP molecule, IL-33 plays a role in modulating inflammation responses in this infection. B6 mice were challenged with a lethal dose of *Orientia* and serially sampled on 2, 6 and 10 days post-infection (dpi). IL-33 expression began to rise at 6 dpi, and the increase was significant at 10 dpi in the kidneys (Fig 1A). Along with this increase, its receptor ST2L also significantly increased at 6 and 10 dpi in the kidneys (Fig 1A). As IL-33 increased as early as 2 dpi in the livers and remained at similar levels throughout the course of infection (Fig 1B), ST2L expression in the liver was also significantly increased at 6 and 10 dpi (Fig 1B). Unlike the kidneys and liver, the lungs had significantly decreased IL-33 expression at 6 and 10 dpi and no statistically significant changes in ST2L expression (Fig 1C). These findings suggest tissue-specific expression of IL-33/ST2L during the infection.

### Disease course of WT and IL-33^-/-^ mice during *Orientia* infection

**Fig 2:**
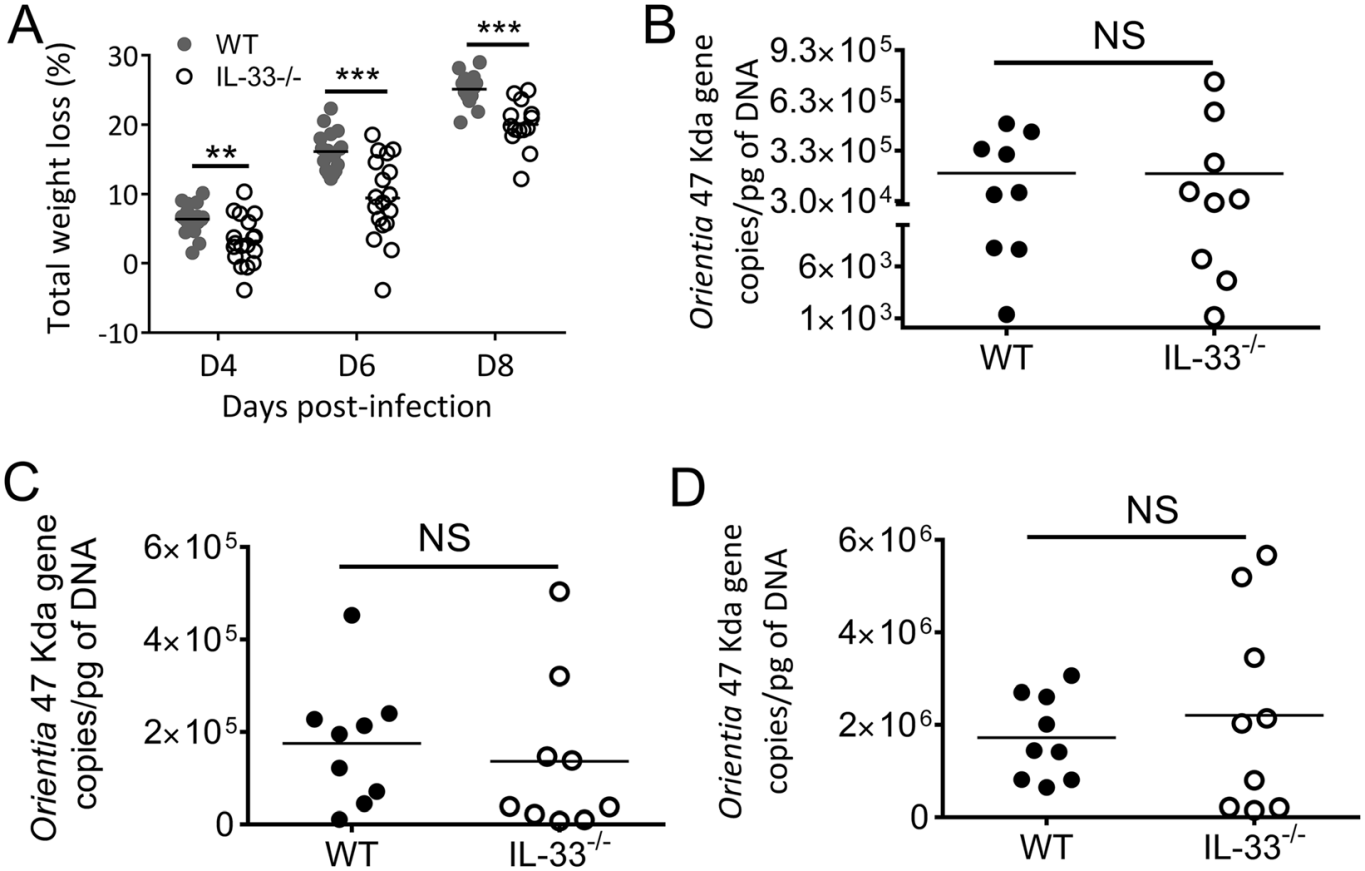
Body weight and tissue bacterial loads of *Orientia*-infected mice. Wild-type (WT) and IL-33^-/-^ mice were lethally challenged with *O*. *tsutsugamushi* Karp strain, as in Fig 1, and monitored daily for whole body weight. Data are presented as total weight loss/mouse (A). Weight loss in WT mice began at 2–3 dpi and continued until they were moribund (9 dpi). IL-33^-/-^ mice had significantly less weight-loss throughout the course of disease and were not moribund at the time of termination of the experiments (9 dpi). Bacterial loads in the kidneys (B), livers (C), and lungs (D) were determined by qPCR. **, *p* < 0.01; ***, *p* < 0.001; NS, no significance.

**Fig 3:**
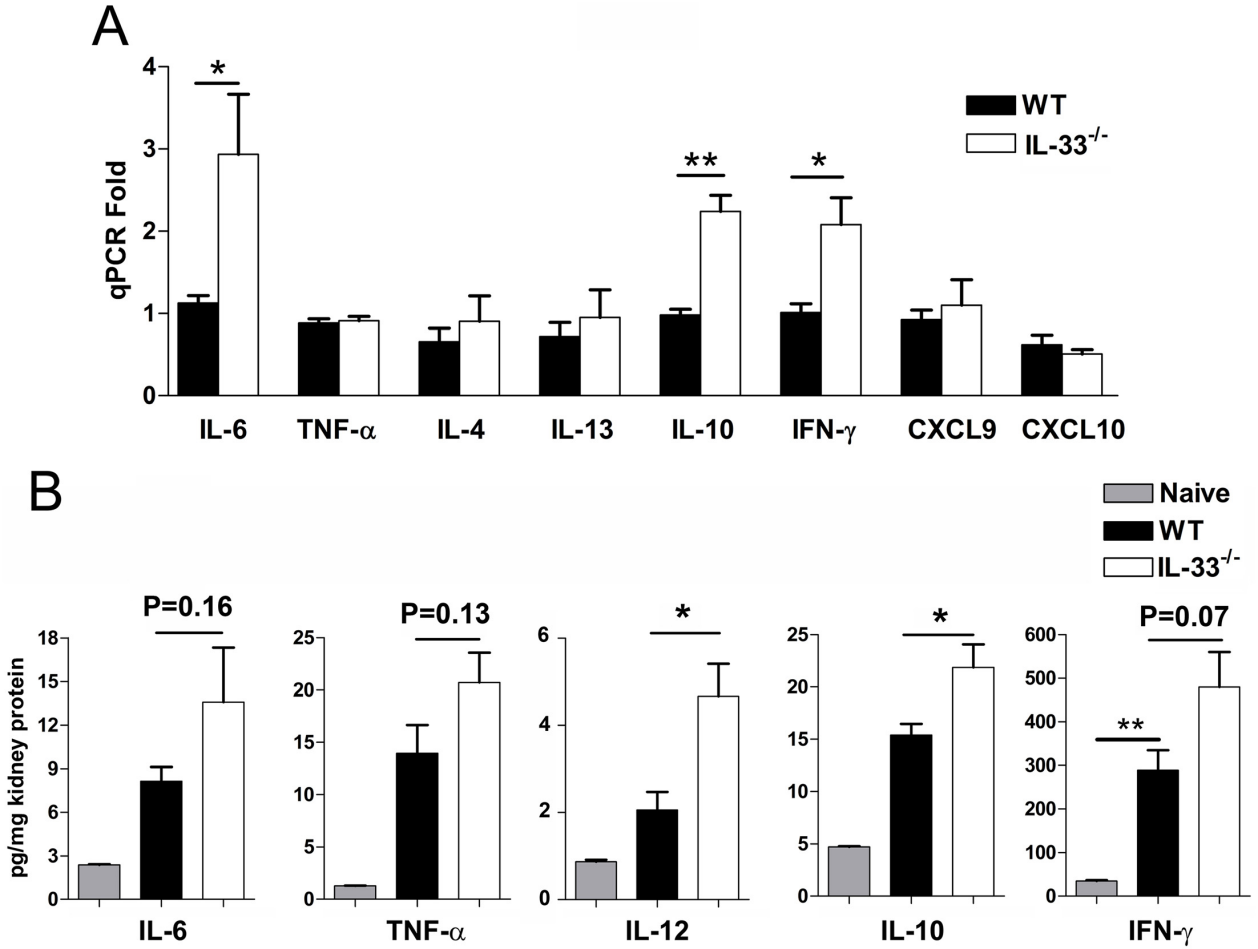
Selective activation of cytokines in the kidneys of infected IL-33^-/-^ mice. WT and IL-33^-/-^ mice were lethally challenged with *O*. *tsutsugamushi* Karp strain, as in Fig 2. (A) Total RNA was extracted from the kidneys at 9 dpi for measuring indicated transcripts by using qRT-PCR. Data are presented as “qPCR fold” (after normalization to the house-keeping genes), and are shown as mean ± SEM in each group. (B) Kidney tissue homogenates were prepared at 9 dpi for measuring protein levels via Bioplex. Representative results are shown from two independent studies with similar trends. *, *p* < 0.05; **, *p* < 0.01; NS, no significance.

To assess the role of IL-33 in scrub typhus progression, we challenged IL-33^-/-^ and WT mice with a lethal dose of *Orientia* and monitored them daily for disease manifestations. IL-33^-/-^ and WT mice both lost body weight from 4 to 8 dpi; however, IL-33^-/-^ mice had significantly less weight loss than did WT mice (Fig 2A) and were considerably more active throughout the course of infection as compared to WT mice. Nevertheless, both groups of mice were moribund at 9 dpi, with similar bacterial loads in the kidneys, livers and lungs (Fig 2B–2D). We also examined a panel of cytokines and chemokines at 9 dpi at the RNA and protein levels (Fig 3). IL-33 deficiency led to significantly higher gene expressions of IL-6, IL-10, and IFN-γ in the kidneys, but no major changes for the expression of Th2 cytokines (IL-4 and IL-13) or chemokines (CXCL9 and CXCL10) (Fig 3A). Similar trends by these cytokines were confirmed in protein levels from the kidneys (Fig 3B). The gene expression levels for IFN-γ, TNF-α, and IL-4 in lung and livers were comparable, while IL-13 expression was undetectable (S1 and S2 Figs).

### Renal histopathology and endothelial apoptosis

**Fig 4:**
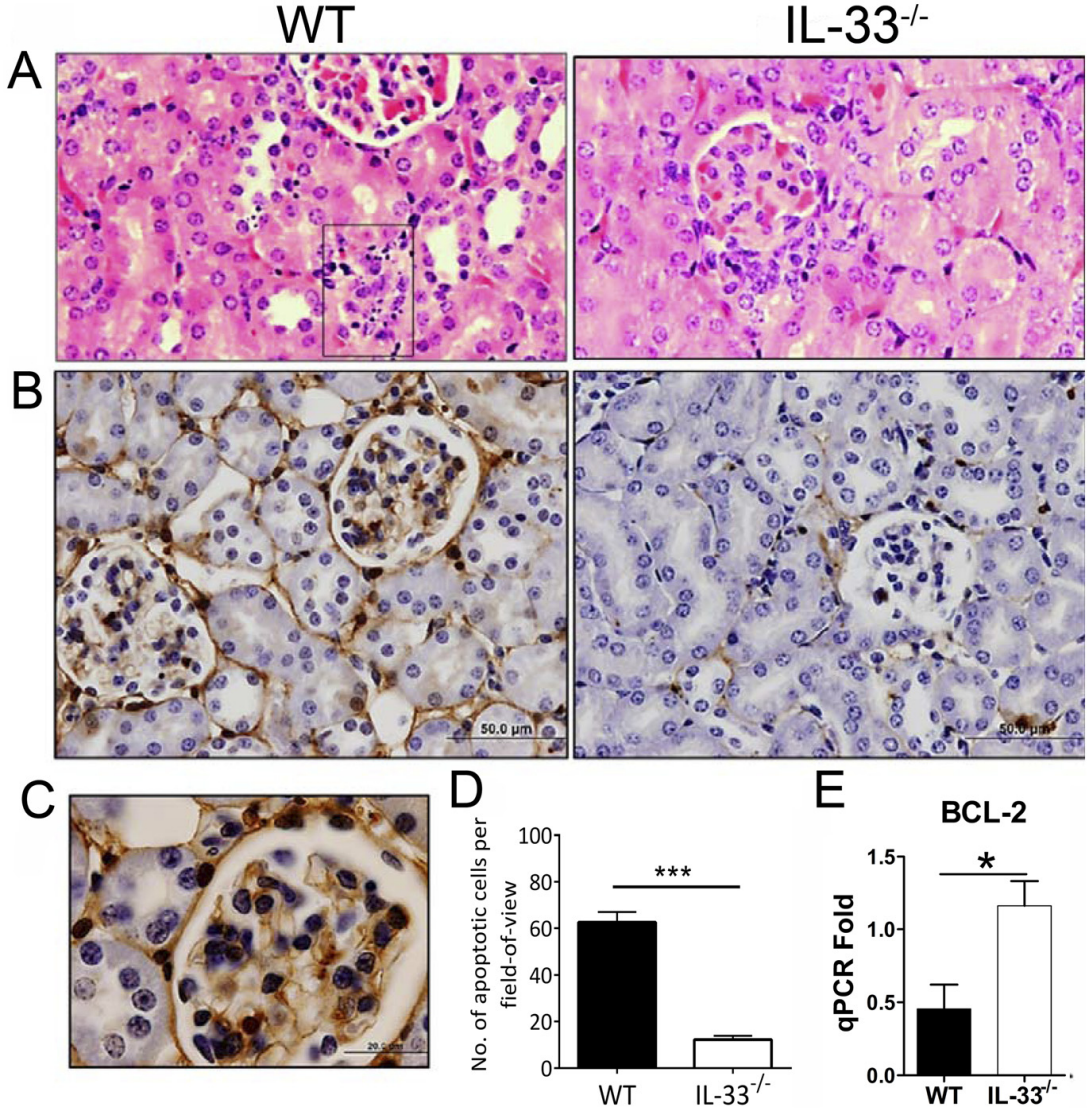
Renal histopathology of *Orientia*-infected mice. WT and IL-33^-/-^ mice were lethally challenged with *O*. *tsutsugamushi* Karp strain, as in Fig 1, and kidney tissues were collected at 9 dpi for analyses. (A) H&E stained sections. Glomerular cellular infiltration and condensed nuclei (in box). (B) Apoptotic cell staining in brown color. Bar = 50 μm. (C) Location of apoptotic cells in the endothelium of WT mice. Bar = 20 μm. (D) The numbers of apoptotic cells were counted for each section. (E) BCL-2 mRNA levels in the kidneys at 9 dpi. *, *p* < 0.05; ***, *p* < 0.001.

IL-33 appears to play a role in pathogenesis of the kidneys [27]. To further gauge inflammatory responses and renal pathology in IL-33^-/-^ mice, we examined and found fewer cellular infiltrates in the kidneys of IL-33^-/-^ mice. Intertubular infiltration was evident, as well as cellular infiltrations in and around the glomeruli in WT mice, whereas IL-33^-/-^ mice had very few instances of infiltration in the kidneys (Fig 4A). A considerable number of dense/fragmented nuclei resembling apoptotic cells were observed in endothelial locations in WT mice (Fig 4A, WT-box). To confirm that these fragmented nuclei were indeed apoptotic cells, TUNEL immunohistochemistry was performed allowing quantification and comparison of apoptotic cell numbers. The kidneys of WT animals had more intense positive staining compared to that in IL-33^-/-^ mice (Fig 4B), especially in the endothelium (Fig 4C). While apoptotic ECs constituted approximately 50% of the total number of apoptotic cells in kidneys from both groups, the WT kidneys had 5-fold more apoptotic cells than did their IL-33^-/-^ counterparts (Fig 4D). To verify the TUNEL findings, we compared the expression of the anti-apoptotic gene BCL-2 and found a significantly higher level of BCL-2 transcripts in IL-33^-/-^ kidneys than seen in WT controls (Fig 4E). There were no major differences of pathology in lungs and livers between infected WT and IL-33^-/-^ mice (S1 and S2 Figs).

### Renal endothelial responses during *Orientia* infection

**Fig 5:**
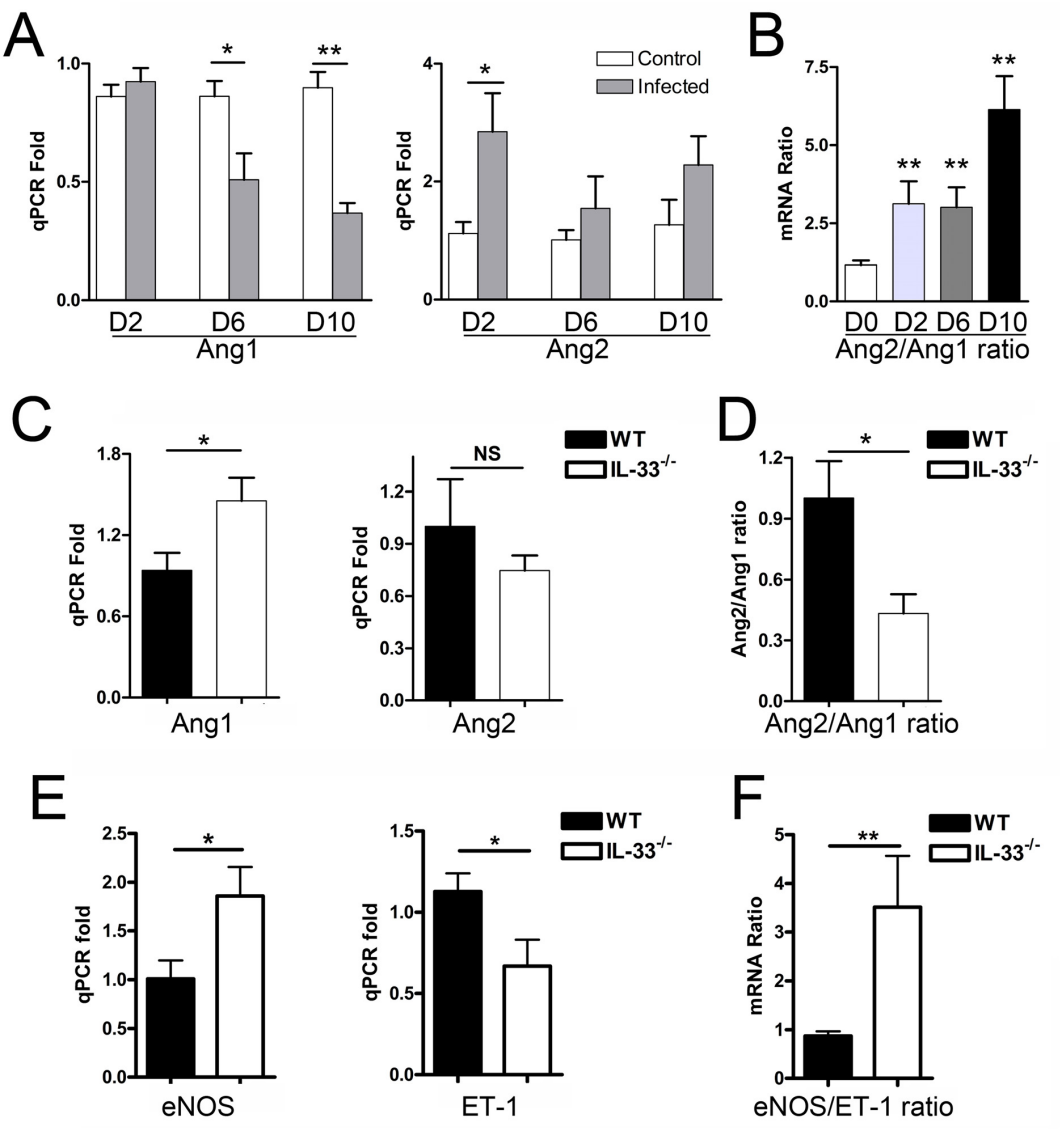
Endothelia cell (EC) activation-associated gene expression in the kidneys of infected mice. WT mice (4-5/group) were inoculated with *O*. *tsutsugamushi* Karp strain as in Fig 1. (A) At 0, 2, 6, and 10 dpi, kidney tissues were analyzed for angiopoietin (Ang) 1 and Ang2 expression by qRT-PCR. Data are presented as “qPCR fold” (after normalization to the housekeeping genes), and are shown as mean ± SEM in each group. (B) The Ang2/Ang1 ratios of individual samples were calculated based on the qRT-PCR data and compared with mock controls (0 dpi). WT and IL-33^-/-^ mice (4-5/group) were infected and euthanatized at 9 dpi. Kidney tissues were analyzed for the expression of Ang1, Ang2 (C), Ang2/Ang1 ratio (D), endothelial nitric oxide synthase (eNOS), Endothlin-1 (ET-1) (E), and eNOS/ET-1 ratio (F), respectively. *, *p* < 0.05; **, *p* < 0.01; NS, no significance.

*Orientia* infection can result in EC stress and activation in lung and liver tissue, as judged by the changes in the Ang2/Ang1 ratio [32]. In the kidneys, elevation in the Ang2/Ang1 ratios were evident as early as 2 dpi and peaked at 10 dpi (Fig 5A and 5B). When EC activation between WT and IL-33^-/-^ mice in the kidneys was compared at 9 dpi (Fig 5C–5E), we found that IL-33^-/-^ mice had significantly attenuated EC stress and activation compared to that in WT controls, as judged by higher levels of Ang1 (an EC-stabilizing factor) and eNOS (a synthase of EC-relaxing factor nitric oxide (NO) [33]), as well as a lower Ang2/Ang1 ratio and lower levels of ET-1 (an important factor in the development of vascular dysfunction by inhibiting eNOS and NO production [34]). The kidney eNOS/ET-1 ratio was significantly higher in the infected IL-33^-/-^ mice (Fig 5F), implying that deficiency in DAMP molecule IL-33 alleviated the endothelial dysfunction in the kidneys of *Orientia*-infected mice. In the liver and lungs, the Ang2/Ang1 ratios were comparable in infected WT and IL-33^-/-^ mice, indicating similar levels of EC activation in these organs (S1 and S2 Figs).

### Administration of rIL-33 increased disease severity, mortality, and vascular dysregulation in the kidneys during the sub-lethal infection

**Fig 6:**
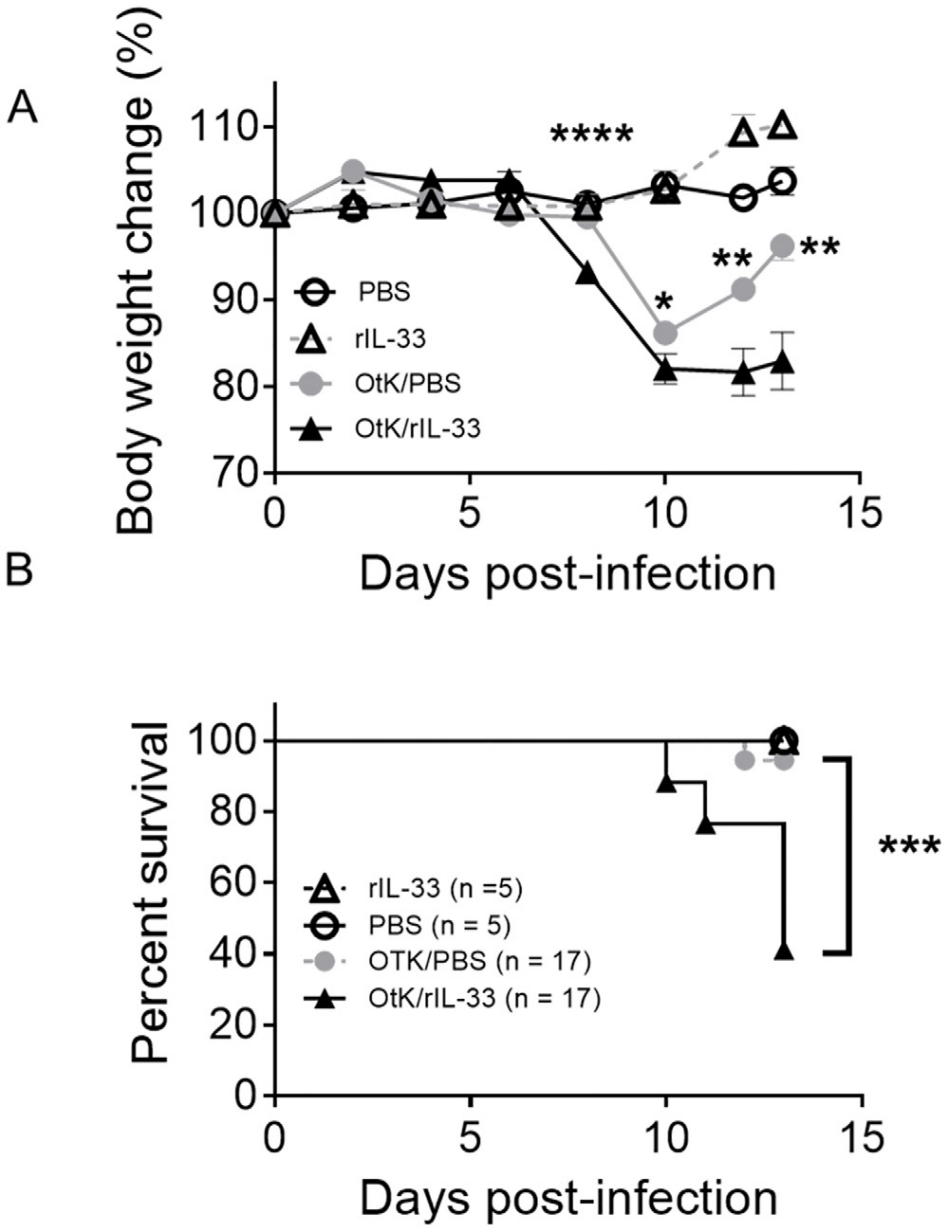
Exacerbated disease and mortality in the presence of exogenous IL-33 during sub-lethal infection. WT mice were infected with sub-lethal dose of *O*. *tsutsugamushi* Karp strain *(OtK)* and then injected with rIL-33 or PBS every other day. Signs of illness were monitored daily. (A) Body weight was monitored daily for 13 days. Shown are representative results from one of the three independent studies with similar trends. (B) Mouse survival was monitored as above. Shown are survival data pooled from three independent experiments. *, *p* < 0.05; **, *p* < 0.01; ***, *p* < 0.001; ****, *p* <0.0001.

**Fig 7:**
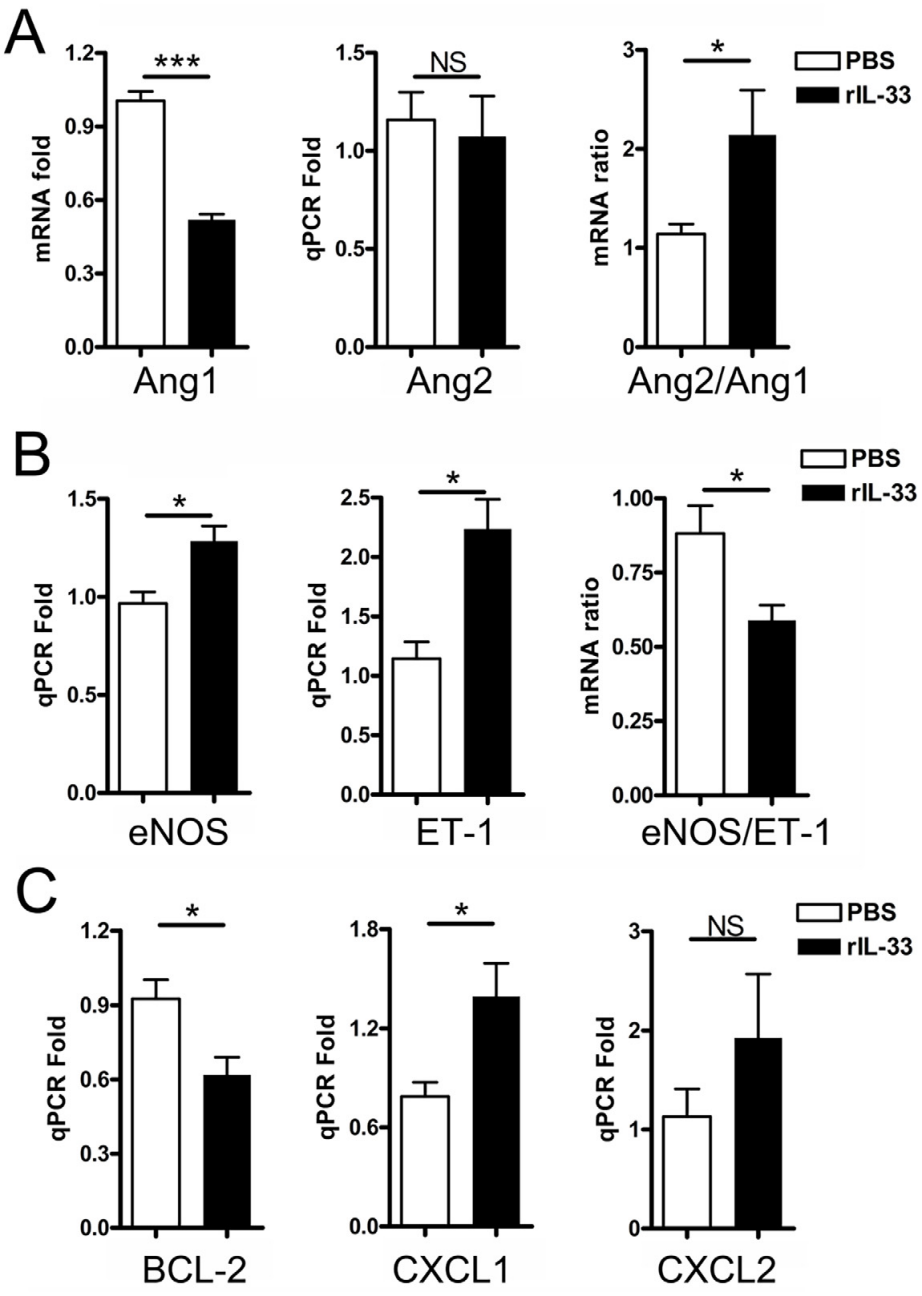
Increased endothelial dysregulation in the kidney of *Orientia*-infected, rIL-33 injected mice. WT mice were treated as in Fig 6. The kidney samples were collected at 9 dpi and analyzed by qRT-PCR for the expression of Ang1, Ang2, Ang2/1 ratios (A), eNOS, Endothlin-1, and their ratios (B), as well as for BCL-2, CXCL1 and CXCL2 (C). Data are presented as “qPCR fold” (after normalization to the house-keeping genes), and are shown as mean ± SEM in each group. Representative results are shown from three independent studies with similar trends. *, *p* < 0.05; ***, *p* < 0.001; NS, no significance.

The above data indicated that the absence of IL-33 during *Orientia* infection resulted in an attenuated weight loss and cellular apoptosis in the kidneys during lethal challenge, but it was not sufficient to increase mouse survival. To validate the function of IL-33, we infected WT mice with a sub-lethal dose of *Orientia* and then i.p. delivered rIL-33 or PBS every other day for 10 days. As shown in Fig 6A, the rIL-33 group lost weight more rapidly starting on 8 dpi than did their PBS-injected counterparts. While the PBS-injected mice recovered part of their body weight after 10 dpi, rIL-33-injected mice exhibited severe signs of disease, with a 64.7% mortality rate (Fig 6B). This increased mortality in the IL-33-injected mice seemed not due to an increase in bacterial loads in the livers or kidneys (S3 Fig). To examine the underlying mechanisms, we examined endothelial markers in the kidneys. We found evidence for increased EC stress and endothelial dysfunction in the kidneys of *Orientia*-infected, IL-33-injected mice, including a significantly reduced Ang1 expression and a near 2-fold increase in Ang2/Ang1 ratio (Fig 7A), which was accompanied with a significantly reduced eNOS/ET-1 ratio (Fig 7B). Exogenous IL-33 also increased the liver inflammation and EC stress, as evidenced by increased liver Ang2/Ang1 ratios (S4 Fig). The down-regulated BCL-2 expression, plus increased CXCL1 expression, in the kidneys of rIL-33-treated mice (Fig 7C) suggested an increased cellular apoptosis and increased IL-33-mediated pro-inflammatory reaction, as previously reported [27].

### *Orientia* infection of human ECs resulted in endothelial activation and induction of IL-33 and its receptors

**Fig 8:**
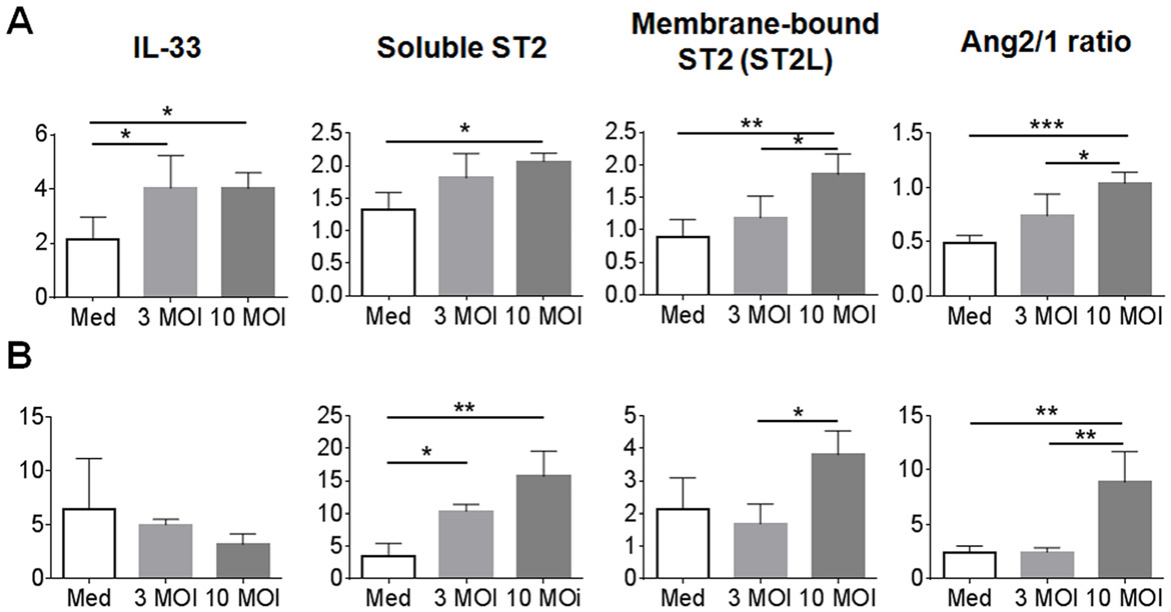
Induced IL-33 and ST2 expression in *Orientia*-infected human umbilical vein endothelial cells (HUVECs). Confluent HUVEC monolayers in 24-well plates were left untreated (Medium only, M) or infected with bacteria at the MOI of 3 or 10. Total RNAs were extracted at 24 h (A) and 48 h (B) of infection for qRT-PCR analyses. Data are presented as “qPCR fold” (after normalization to the house-keeping genes), and are shown as mean ± SEM in each group. For each sample, mRNA levels of IL-33, ST2 receptor isoforms (sST2 and ST2L), Ang1 and Ang2 were measured. The Ang2/1 ratios were calculated, as in Fig 4. *, *p* < 0.05; **, *p* < 0.01; ***, *p* < 0.001; NS, no significance.

To further examine the role of EC stress, we infected human umbilical vein endothelial cells (HUVEC) in vitro at 3 and 10 multiples of infection (MOI), respectively. At 24 hours post-infection (hpi), we found an infectious dose-dependent increase in the expression of IL-33, soluble ST2 (sST2), membrane-bound ST2L, and the Ang2/Ang1 ratio (Fig 8A). At 48 hpi, IL-33 levels were similar to those in controls, but elevation in sST2, ST2L, and Ang2/Ang1 ratio remained significant, especially for high-dose infection groups (Fig 8B). We also examined the secretion of IL-33 proteins in culture supernatants of infected HUVECs (MOI 3 and MOI 10) at 0, 3, 24 and 48 hpi by using an ELISA assay. Appreciable IL-33 was detected among high-dose infection groups, rather than in samples infected with 3 MOI and control samples (S5 Fig). Our *in vitro* data were consistent with those from mouse studies *in vivo*, implying an important role for IL-33/ST2-mediated responses during *Orientia* infection.

## Discussion

**Fig 9:**
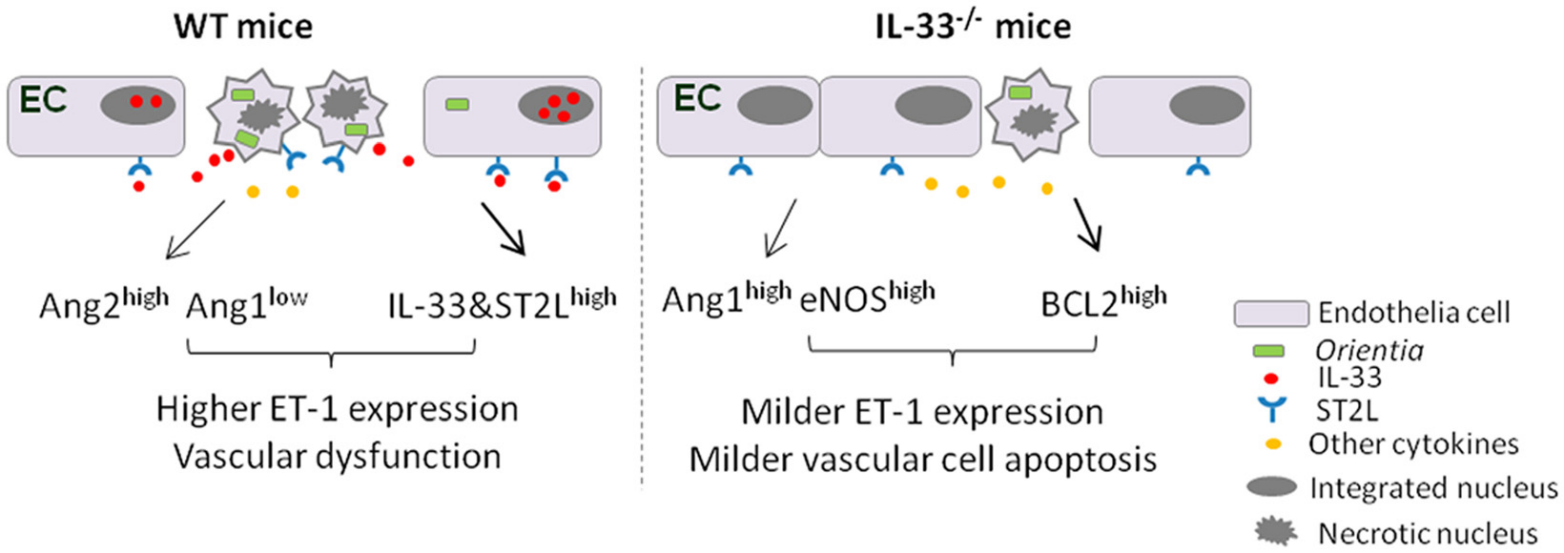
A schematic illustration of EC responses in *Orientia*-infected WT versus IL-33^-/-^ mice. In WT mice, *O*. *tsutsugamushi* infection increases IL-33 and ST2L expression in ECs, and triggers IL-33 release from the nucleus of stressed or activated ECs. The binding of IL-33 to its membrane-bound receptor ST2L can promote EC activation and apoptosis, as judged by altered expression of Ang proteins. These events collectively contributed to increased ET-1 expression, decreased EC integrity, and increased vascular damage. In the absence of IL-33/ST2L signaling, EC stress and apoptosis are attenuated, partially due to relatively higher Ang1, eNOS, and BCL2 levels, but relatively lower ET-1 levels. The preserved EC integrity, together with the production of other immune cytokines facilitates tissue healing and host survival.

Invasion of *O*. *tsutsugamushi* can cause acute tubular necrosis, leading to renal failure in patients [35, 36]; however, the underlying mechanism is unclear. The DAMP molecule IL-33 is known to be a potent endothelial activator, promoting angiogenesis and vascular permeability [37], and can also selectively target the non-quiescent ECs, driving pro-inflammatory cell activation [38]. IL-33 also contributes to the pathogenesis of cisplatin-induced acute kidney injury [27]. However, it is unclear whether IL-33 modulates tissue injury and progression of scrub typhus. Here, we demonstrate that there was a significant increase in IL-33 and its ST2L receptor expression in the kidneys and liver during *O*. *tsutsugamushi* infection in mice and that IL-33 contributed to renal pathology and endothelial damage during experimental scrub typhus infection. The absence of IL-33 signaling during lethal infection attenuated cellular and tissue damage and delayed the onset of disease (i.e. weight loss), although such changes were not sufficient to rescue the mice from death. Conversely, addition of exogenous IL-33 during a sub-lethal infection exacerbated the activation of renal endothelium and lethality. Moreover, *Orientia* infection alone was capable of inducing gene expression of IL-33 and its receptors, as well as endothelial activation, in human endothelial cells. We have proposed a pathogenic role of IL-33 in endothelial dysregulation during the infection (Fig 9). This is the first study to address the role of IL-33 in a mouse model of scrub typhus.

IL-33 has crucial and diverse roles in infectious diseases, depending on the type of infectious agents, acute or chronic infection stages, tissues involved, and host immune microenvironments [39]. The protective roles of the IL-33/ST2 axis have been reported during chronic viral infection in the liver, via promoting CD8^+^ T-cell responses [40], repressing inflammatory cytokine TNF-α, inducing type 2 innate lymphoid cells (ILC2), and protecting the liver in acute adenovirus infection [30]. IL-33-induced ILC2 also promotes lung tissue homeostasis in influenza virus infection [41]. However, IL-33 also plays deleterious roles during *Cryptococcus neoformans*-induced lung mycosis and allergic inflammation in the lungs [42, 43]. Research concerning the role of IL-33 in kidney infection is relatively limited, and a few reports are focused on cisplatin or *Candida albicans*-induced renal injury [13, 44]. Our data demonstrate that infection with *O*. *tsutsugamushi* Karp strain can increase gene expression of IL-33 and ST2L in the kidneys and liver (Fig 1). The cellular sources of IL-33 in *Orientia*-infected tissues was not examined in this study, due to technical issues with respect to cell isolation in the ABSL3 facility; but the possible candidates may include ECs in the kidneys [22] and hepatocytes in the liver [45]. The marked reduction of IL-33 expression in the lung tissues at 6 and 10 dpi may not be surprising, given the massive cellular necrosis and tissue damage [32]. Yet, the tendency of reduced IL-33 expression at 2 dpi in the lung was interesting. Regardless of the underlying mechanisms, our data suggest tissue-specific roles of endogenous IL-33 and highlight its contributions in renal injury, cellular apoptosis, and endothelial activation in mouse model of severe scrub typhus.

To further investigate how IL-33 regulates immune responses and why IL-33^-/-^ mice have attenuated weight loss and kidney injury (Figs 2–4), we examined a panel of immune cytokines in the kidney after infection. We have demonstrated previously that *Orientia* infection induces strong type 1, but impaired, type 2 immune responses, in several tissues [32]. In the present study, we found that IL-33 deficiency or exogenous rIL-33 did not drastically change type 1 or type 2 cytokine expression during *Orientia* infection, as reported in a *C*. *albicans*-induced renal injury model [44]. While IL-33 did not reprogram type 1 vs. type 2 responses during *Orientia* infection, the IL-33/ST2 signaling significantly amplified the magnitude of pro-inflammatory responses (Fig 3), cellular apoptosis, EC stress and activation, and host death (Figs 4–7). On one hand, we observed the upregulated CXCL1, but decreased anti-apoptotic gene BCL-2, in the kidneys by rIL-33 treatment. This finding is similar to that in the cisplatin-induced renal failure model [27], suggesting the unique mechanism that IL-33/CXCL1 axis may play a critical role in renal injury not only in the toxic reagent-induced model but also in infectious diseases. On the other hand, we found that IL-33^-/-^ mice have a higher expression of anti-inflammatory cytokine IL-10 at both the gene and protein levels in *Orientia* infection. This increased IL-10 may play a role in renal protection in infected IL-33^-/-^ mice [46]. Some acute inflammatory mediators (e.g. IL-6, IL-12 and IFN-γ) may contribute to bacterial control [47, 48].

*Orientia* infection *in vitro* can activate ECs, leading to cell apoptosis [49, 50]. ECs are known to be the source of nucleus IL-33 [22]; however, few studies have focused on the interaction and DAMP molecule expression in ECs infected with *Orientia*. We have provided evidence that *Orientia* infection *in vitro* increased IL-33 and ST2L expression in and the activation of human ECs by 24 h. Prolonged stimulation (48 h), however, did not alter the IL-33 gene expression levels, but dramatically increased both the soluble and membrane-bound ST2 forms of receptors (Fig 8); this may partially explain our difficulty in detecting IL-33 proteins in the culture supernatants. Since sST2 can bind IL-33 and block intracellular IL-33/ST2L signaling [51], this increased sST2 level may counterbalance the excessive IL-33 signal and keep the homeostasis. In addition to being the source of IL-33, ECs are the target of IL-33 [38]. It was previously shown that angiogenesis in ECs was induced by stimulating endothelial NO production via the ST2/TRAF6-Akt-eNOS signaling pathway [37]. It will be interesting to further examine the intracellular signaling events that regulate IL-33/ST2 expression during *O*. *tsutsugamushi* infection.

Based on our *in vitro* studies in human ECs and *in vivo* studies in WT and IL-33^-/-^ mice, we have proposed a pathogenic role of IL-33 in endothelial dysregulation during the infection (Fig 8). High-dose *O*. *tsutsugamushi* infection in ECs and other cell types can trigger EC stress, dysfunction, and apoptosis. In the WT mice, the increased IL-33 and ST2 expression on EC and IL-33 production may further exacerbate EC stress and damage. These IL-33/ST2-mediated effects are diminished or markedly reduced in *O*. *tsutsugamushi*-infected IL-33^-/-^ mice, leading to attenuated renal endothelium activation but higher levels of Ang1 in the kidneys. We have provided evidence that endogenous IL-33 promotes EC inflammation during *Orientia* infection, via multiple mechanisms, which includes reduced Ang1 and eNOS expression, but increased Ang2 and ET-1 expression in the infected kidneys, as in reports for other models [37, 52]. The interplays among Ang1, eNOS and ET-1 in *Orientia*-infected ECs warrant further investigation [34]. As expected, our results reveal that IL-33 regulates the balance of Ang1/Ang2 as well as that of eNOS/ET-1, modulating the EC inflammation and tissue dysregulation in the kidneys during severe scrub typhus. Our findings are important in the context of a recent report, showing that IL-33 concentrations in human serum strongly correlated with the severity of Hantaan infection, another endotheliotropic pathogen [53]. Therefore, while IL-33 plays a protective role in other models such as viral hepatitis, it has a pathogenic role in endotheliotropic diseases.

Overall, this study indicates a significant role of IL-33 alarmin in endothelial activation and renal damage, highlighting infection-triggered EC damage and IL-33-mediated pathological changes during the course of *O*. *tsutsugamushi* infection. This study provides a better understanding of the pathogenesis and a potential biomarker for monitoring disease progression of scrub typhus cases.

## Materials and Methods

### Mouse infection and ethics statement

Female WT B6 mice were purchased from Jackson Laboratory. IL-33^-/-^ mice on the B6 background were kindly provided by Dr. Rene de Waal Malefyt (Merck, Palo Alto, CA). Mice were maintained under specific pathogen-free conditions and used at 8- to 12 weeks of age following protocols approved by the Institutional Animal Care and Use Committee (protocol # 1302003) at the University of Texas Medical Branch (UTMB) in Galveston, TX. All mouse infection studies were performed in the ABSL3 facility in the Galveston National Laboratory located at UTMB; all tissue processing and analysis procedures were performed in the BSL2 or BSL3 facilities. All procedures were approved by the Institutional Biosafety Committee, in accordance with Guidelines for Biosafety in Microbiological and Biomedical Laboratories. UTMB operates to comply with the USDA Animal Welfare Act (Public Law 89–544), the Health Research Extension Act of 1985 (Public Law 99–158), the Public Health Service Policy on Humane Care and Use of Laboratory Animals, and the NAS Guide for the Care and Use of Laboratory Animals (ISBN-13). UTMB is a registered Research Facility under the Animal Welfare Act and has a current assurance on file with the Office of Laboratory Animal Welfare, in compliance with NIH Policy.

*O*. *tsutsugamushi* Karp strain was used herein, and all infection studies were performed with the same bacterial stock prepared from liver extracts pooled from several infected mice. Infectious organisms were then quantified via a focus forming assay as described previously [31, 32]. WT and IL-33^-/-^ mice were inoculated intravenously (*i*.*v*.) with a lethal dose of *O*. *tsutsugamushi* (4.5 x 10^6^ FFU in 200 μl). Control mice were similarly injected with PBS. At 9 dpi, serum and tissue samples were collected and inactivated for subsequent analyses. To study the effect of excess IL-33 mice received a mostly sub-lethal injection of 8.5 x 10^5^ organisms. After infection mice were injected intraperitoneally with either PBS or 1 μg of rIL-33 at 2, 4, 6, 8, and 10 dpi, respectively. Animals were monitored for signs of disease progression daily until the end of the experiment (13 dpi).

### Cell culture and infection

HUVECs were cultured as described previously [54]. Briefly, HUVECs (Cell Application, San Diego, CA) were cultivated in Prigrow I medium supplemented with 10% (vol/vol) heat-inactivated FBS in 5% (vol/vol) CO_2_ at 37°C. All experiments were performed between passages 5 and 7, and cells were maintained in Prigrow I medium with 3% (vol/vol) FBS. When HUVECs were confluent, they were collected and seeded onto 24-well plates (Corning Inc., Corning, NY). Once all wells were confluent, the HUVEC monolayers were infected with either 3 MOI, 10 MOI, or media only. Total RNA was extracted from each plate at 3, 24, and 48 hours post-infection (hpi) by using an RNeasy mini kit (Qiagen, Valencia, CA) and digested with RNase-free DNase (Qiagen). Gene expression was determined as described below. Cell-free culture supernatants were collected and stored in -80°C until protein analysis.

### Enzyme-linked immunosorbent assay (ELISA)

IL-33 concentrations in supernatants of control and infected HUVECs were determined by using human IL-33 Quantikine ELISA kits (R&D Systems, Minneapolis, MN) following the manufacturer’s protocol. Briefly, 100 μl of supernatant was added to each well of the anti-hIL-33-coated, 96-well ELISA plate. The plate was analyzed using a Versamax Turntable Microplate Reader (Molecular Devices, Sunnyvale, CA) and Softmax Pro V.4.0. All procedures were performed in the BSL3 facility.

### Quantitative reverse transcriptase PCR (qRT-PCR) analysis

Mouse tissues were collected in an RNA*Later* solution (Ambion, Austin, TX) at 4°C overnight to inactivate infectious bacteria and stored at -80°C for subsequent analyses. Total RNA was extracted from tissue by using an RNeasy mini kit (Qiagen, Valencia, CA) and digested with RNase-free DNase (Qiagen). cDNA was synthesized with the iScript cDNA synthesis kit (Bio-Rad Laboratories, Hercules, CA). The abundance of target genes was measured by qRT-PCR by using a Bio-Rad CFX96 real-time PCR apparatus, and a SYBR Green Master mix (Bio-Rad) was used for all PCR reactions. PCR reactions were started at 95°C for 3 min, followed by 39 cycles of 95°C for 10 sec, and 60°C for 10 sec, and ended with an elongation step at 72°C for 10 sec. Dissociation melting curves were obtained after each reaction to confirm the purity of PCR products. Relative abundance of mRNA expression was calculated by using the 2^-ΔΔCT^ method. Glyceraldehyde-3-phosphate dehydrogenase (GAPDH) and β-actin were used as the housekeeping genes. Primer sequences are listed in S1 Table.

### Bacterial load determination

Bacterial loads were assessed by quantitative real-time PCR as described previously [31, 32]. DNA was extracted by using a DNeasy Kit (Qiagen, Gaithersburg, MD) from the tissue samples, and the bacterial load at each time point and for each organ sampled was determined by quantitative real-time PCR. The gene for a 47-kDa protein was amplified by using specific primers (OtsuF630 and OtsuR747 (IDT, Coralville, IA). PCR products were detected with a specific probe OtsuPr665 (Applied Biosystems, Foster City, CA). Bacterial loads were normalized to total nanogram (ng) of DNA per μL for the same sample, and data are expressed as the gene copy number of 47-kDa protein per picogram (pg) of DNA. 47-kDa gene copy number was determined by known concentrations of control plasmid containing single-copy inserts of the gene. The plasmid concentration was determined and serially diluted 10-fold for the standards.

### Apoptotic cell staining and cell counts

All tissues were fixed in 10% neutral-buffered formalin and embedded in paraffin, and sections (5-μm thickness) were stained with hematoxylin and eosin. Apoptosis was detected by using a Millipore ApopTag Peroxidase *In Situ* Apoptosis Detection kit. Kidneys were assessed for positive staining; five, 40x images were taken on an Olympus BX53 microscope. Images were used so that multiple observers could assess the same fields for apoptosis. DAB-positive cells were counted as apoptotic and divided into endothelial cells, based on cellular and nuclear morphology, and other cells. Cells that were rounded or otherwise not recognizable as ECs were counted as other cells. The number of apoptotic cells was counted per 40x field-of-view. The observers’ counts were pooled and averaged and then numbers compared as total number of apoptotic cells per view and apoptotic ECs only.

### Bio-Plex assay

Cytokine profiles in the tissues were characterized by using Procarta Plex Mouse Cytokine Panel (eBioscience, San Diego, CA). Briefly, kidney protein was extracted by using RIPA (Cell Signaling Technology, Danvers, MA) plus Protease Inhibitor Cocktails (Sigma, St. Louis, MO). The concentration of protein was determined by a Pierce BCA Protein Assay kit (Thermo Scientific, Waltham, MA). Colored magnetic beads coated with different antigens were mixed together with kidney protein samples, and then allowed to incubate for overnight at 2–8°C. After three wash cycles, detection antibody was added and allowed to incubate for 1 h at room RT, followed by incubation with Streptavidin-Phycoerythrin for 30 min at RT. After removal of excess conjugate, 150 μl of sheath fluid was added to each well. The beads were read on a Bio-Rad Bio-Plex 200 System. Raw data were measured as the relative fluorescence intensity and then converted to the concentration according to the standard curve.

### Statistical analysis

Data were presented as mean ± standard errors of the mean (SEM). Differences between individual treatment and control groups were determined by using Student’s t test. One-way ANOVA was used for multiple group comparisons. Statistically significant values are referred to as *, *p* < 0.05; **, *p* < 0.01, ***, p < 0.001, ****, p < 0.0001; NS, no significance.

## Supporting Information

S1 Table Real-time PCR primers of the tested genes. The primer sequences for genes analyzed in this study are listed (5’ to 3’ direction).
(DOCX)

S1 Fig Immune responses and pathological changes in the lungs of IL-33^-/-^ mice. WT mice (black bars) and IL-33^**-/-**^ mice (open bars) were inoculated i.v. with *O*. *tsutsugamushi* Karp stain (4.5 x 10^6^ FFU, 4-5/group). (A-C) At 0 or 9 dpi, total RNA was extracted from lung tissues for qRT-PCR analyses of indicated markers. Data are shown as mean ± SEM in each group and presented as “qPCR fold” (after normalization to the house-keeping genes). Representative results are shown from two independent studies with similar trends. *, *p* < 0.05. ND, not detected. NS, not significant. At 9 dpi, pulmonary pathology was similar in WT mice (D) and IL-33 KO (E), consisting of diffuse cellular infiltrates, alveolar septa thickening, and pulmonary edema. Bar = 200 μm. There were no major differences in apoptotic staining (brown) in infected WT mice (F) and IL-33 KO (G); Bar = 50 μm.
(TIF)

S2 Fig Immune responses and pathological changes in the livers of IL-33^-/-^ mice. WT and IL-33^**-/-**^ mice (4-5/group) were inoculated i.v. with *O*. *tsutsugamushi* Karp stain (4.5 x 10^6^ FFU). (A-C) At 0 and 9 dpi, total RNA was extracted from liver for qRT-PCR analyses of effector cytokines. Data are shown as mean ± SEM in each group and presented as “qPCR fold” (after normalization to the house-keeping genes). Representative results are shown from two independent studies with similar trends. ND, not detected. NS, not significant. At 9 dpi, hepatic pathology was similar in WT mice (D) and IL-33 KO (E), consisting of diffuse and focal cellular infiltrates as well as vasculitis. Bar = 200 μm. There were no major differences in apoptotic staining (brown) in infected WT mice (F) and IL-33 KO (G); Bar = 50 μm.
(TIF)

S3 Fig Bacterial loads of tissues from *Orientia*-infected mice injected with PBS or rIL-33. WT mice were infected with sub-lethal dose of *O*. *tsutsugamushi* Karp strain and then injected with rIL-33 or PBS every other day, as described in Fig 6. The kidneys (A) and livers (B) were collected from moribund mice (between 10–12 dpi for some rIL-33-treated mice) and terminated mice (13 dpi for the rest mice) for the analysis of tissue bacterial loads by qPCR. NS, no significance.
(TIF)

S4 Fig Increased endothelial dysregulation in the livers of *Orientia*-infected, rIL-33-injected mice. WT mice were infected and treated as in Fig 6. The liver samples were collected at 0 and 9 dpi and analyzed by qRT-PCR for the expression of Ang1, Ang2, and Ang2/1 ratios (A), as well as eNOS, Endothlin-1, and their ratios (B). Data are presented as “qPCR fold” (after normalization to the house-keeping genes), and are shown as mean ± SEM in each group. Representative results are shown from three independent studies with similar trends. *, *p* < 0.05; NS, no significance.
(TIF)

S5 Fig IL-33 protein levels in the supernatants of *Orientia*-infected HUVEC cells. Confluent HUVEC monolayers in 24-well plates were left untreated (Med) or infected with bacteria either at MOI of 3 or MOI of 10, as described in Fig 8. Supernatants were collected from two independent experiments at 3, 24, and 48 hpi and analyzed for IL-33 secretion by using Quantikine ELISA kits. Data are presented as mean ± SEM. There were no significant differences among the infected and control groups.
(TIF)
