# Supplementary material for: IL-33-Dependent Endothelial Activation Contributes to Apoptosis and Renal Injury in Orientia tsutsugamushi-Infected Mice
Source: PLoS Negl Trop Dis. 2016 Mar 4;10(3):e0004467. doi: 10.1371/journal.pntd.0004467 (PMC4778942; doi:10.1371/journal.pntd.0004467)
Supplement: S1 Table — The primer sequences for genes analyzed in this study are listed (5’ to 3’ direction). (DOCX) [file pntd.0004467.s001.docx]

| **Supplemental Materials** |
| --- |

**Table. S1 Real-time PCR primers of genes**

Forward (5’ to 3’) Reverse (5’ to 3’)

**Mouse**

Ang1 ATCCCGACTTGAAATACAACTGC CTGGATGATGAATGTCTGACGAG

Ang2 GGTTGCTATCCGTAAAGAAGAGC GGGGAAGGTCAGTGTGTAGATG

IFN-γ ATGAACGCTACACACTGCATC CCATCCTTTTGCCAGTTCCTC

IL-4 GGTCTCAACCCCCAGCTAGT GCCGATGATCTCTCTCAAGTGAT

IL-6 TAGTCCTTCCTACCCCAATTTCC TTGGTCCTTAGCCACTCCTTC

IL-10 GCTCTTACTGACTGGCATGAG CGCAGCTCTAGGAGCATGTG

IL-13 CCTGGCTCTTGCTTGCCTT GGTCTTGTGTGATGTTGCTCA

IL-33 TCCAACTCCAAGATTTCCCCG CATGCAGTAGACATGGCAGAA

ST2L TGTATTTGACAGTTACGGAGGGC ACTTCAGACGATCTCTTGAGACA

TNF-α ATAGCTCCCAGAAAAGCAAGC CACCCCGAAGTTCAGTAGACA

eNOS CCAGTGCCCTGCTTCATC GCAGGGCAAGTTAGGATCAG

BCL2 ATGCCTTTGTGGAACTATATGGC GGTATGCACCCAGAGTGATGC

CXCL1 CTGGGATTCACCTCAAGAACATC CAGGGTCAAGGCAAGCCTC

CXCL2 CCAACCACCAGGCTACAG GCGTCACACTCAAGCTCTG

Endothelin1 GCACCGGAGCTGAGAATGG GTGGCAGAAGTAGACACACTC

GAPDH TGGAAAGCTGTGGCGTGAT TGCTTCACCACCTTCTTGAT

**Human**

IL-33 GTGACGGTGTTGATGGTAAGAT AGCTCCACAGAGTGTTCCTTG

ST2L AGCAGAGTGGCCTCAATCCA TGGAACCACACTCCATTCTGC

Ang1 AGAACCTTCAAGGCTTGGTTAC GGTGGTAGCTCTGTTTAATTGCT

Ang2 CTCGAATACGATGACTCGGTG TCATTAGCCACTGAGTGTTGTTT

sST2 CTGTCTGGCCCTGAATTTGC TGGAACCACACTCCATTCTGC

GAPDH ACAACTTTGGTATCGTGGAAGG GCCATCACGCCACAGTTTC

***Orientia***

OtsuF630 AACTGATTTTATTCAAACTAATGCTGCT

OtsuR747 TATGCCTGAGTAAGATACGTGAATGGAATT

OtsuPr665 Probe FAM-TGGGTAGCTTTGGTGGACCGATGTTTAATCT-TAMRA

**Table S1. Real-time PCR primers of genes.** The primer sequences for genes analyzed in this study are listed (5’ to 3’ direction).
