# Supplementary figures and images for: IL-33-Dependent Endothelial Activation Contributes to Apoptosis and Renal Injury in Orientia tsutsugamushi-Infected Mice
Source: PLoS Negl Trop Dis. 2016 Mar 4;10(3):e0004467. doi: 10.1371/journal.pntd.0004467 (PMC4778942; doi:10.1371/journal.pntd.0004467)

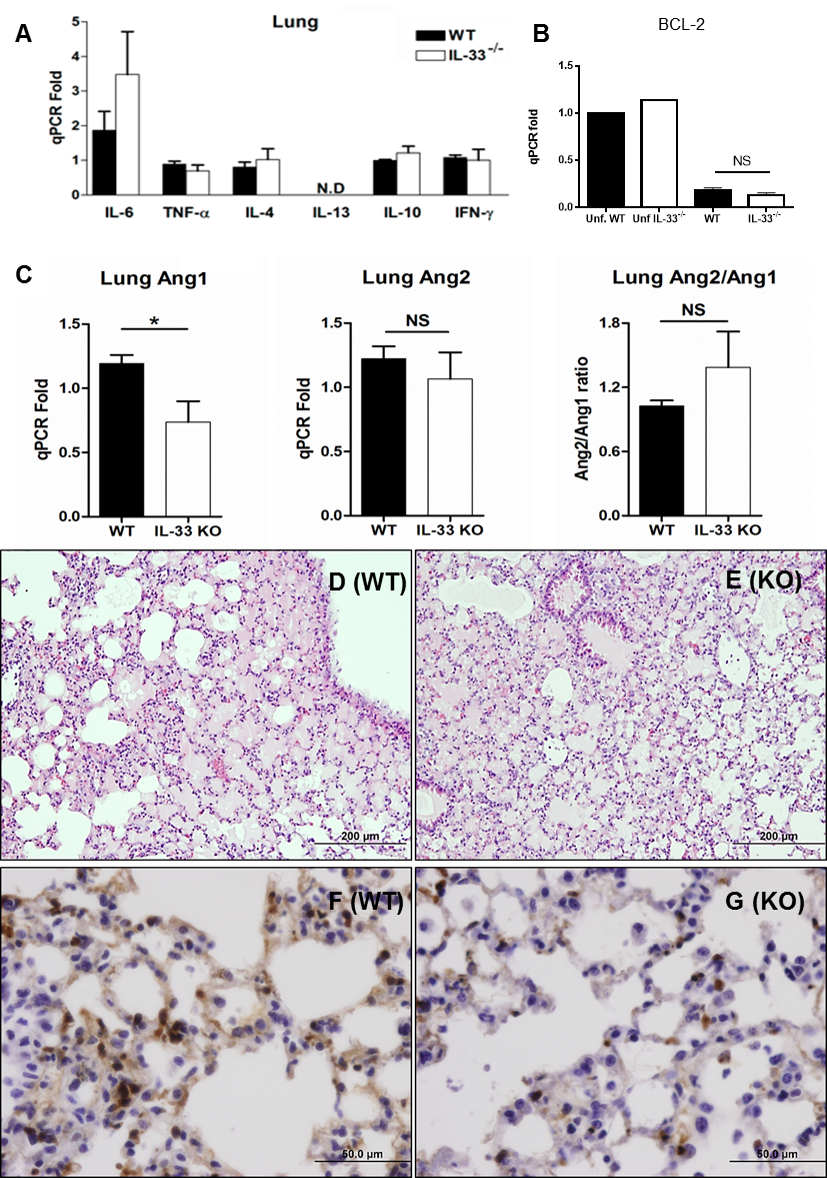

Supplement: S1 Fig — WT mice (black bars) and IL-33-/- mice (open bars) were inoculated i.v. with O. tsutsugamushi Karp stain (4.5 x 10^6^ FFU, 4-5/group). (A-C) At 0 or 9 dpi, total RNA was extracted from lung tissues for qRT-PCR analyses of indicated markers. Data are shown as mean ± SEM in each group and presented as “qPCR fold” (after normalization to the house-keeping genes). Representative results are shown from two independent studies with similar trends. *, p < 0.05. ND, not detected. NS, not significant. At 9 dpi, pulmonary pathology was similar in WT mice (D) and IL-33 KO (E), consisting of diffuse cellular infiltrates, alveolar septa thickening, and pulmonary edema. Bar = 200 μm. There were no major differences in apoptotic staining (brown) in infected WT mice (F) and IL-33 KO (G); Bar = 50 μm. (TIF) [file pntd.0004467.s002.tif]

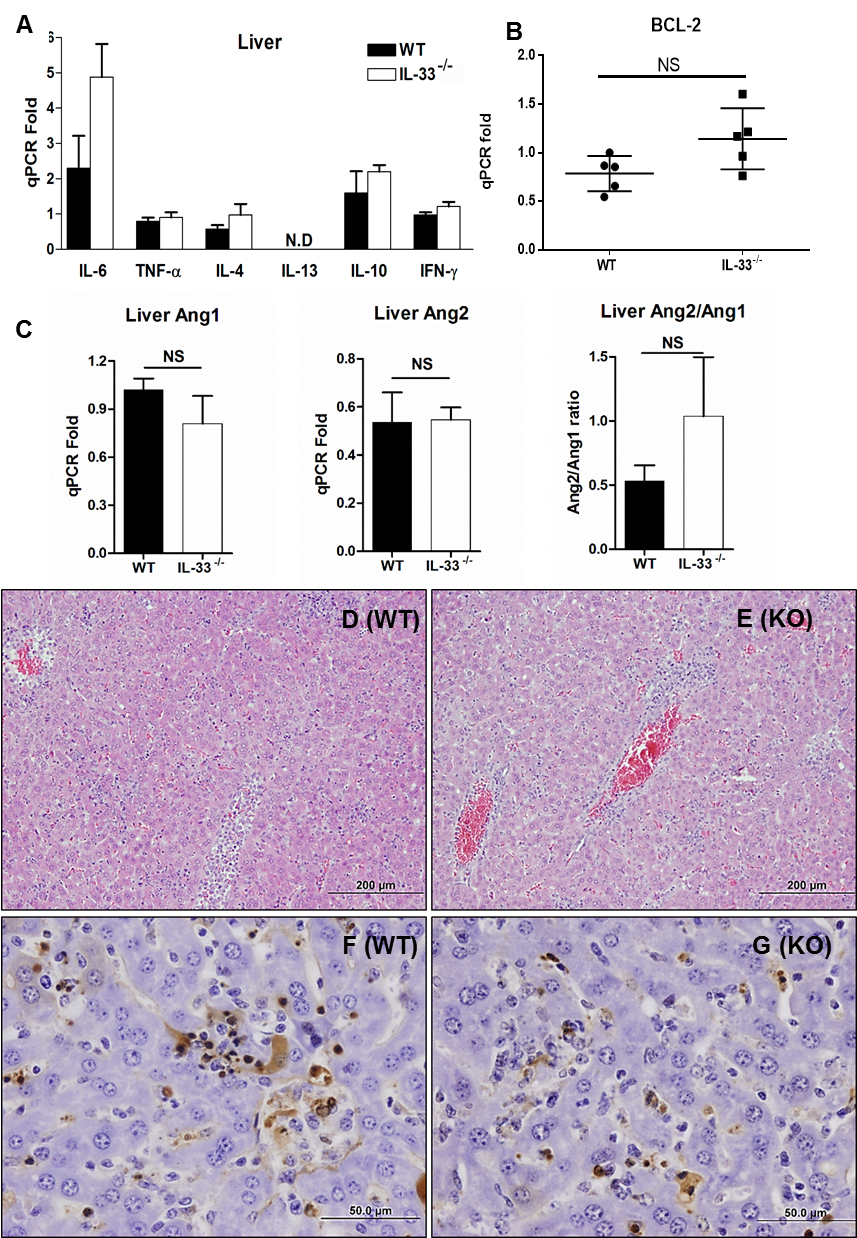

Supplement: S2 Fig — WT and IL-33-/- mice (4-5/group) were inoculated i.v. with O. tsutsugamushi Karp stain (4.5 x 10^6^ FFU). (A-C) At 0 and 9 dpi, total RNA was extracted from liver for qRT-PCR analyses of effector cytokines. Data are shown as mean ± SEM in each group and presented as “qPCR fold” (after normalization to the house-keeping genes). Representative results are shown from two independent studies with similar trends. ND, not detected. NS, not significant. At 9 dpi, hepatic pathology was similar in WT mice (D) and IL-33 KO (E), consisting of diffuse and focal cellular infiltrates as well as vasculitis. Bar = 200 μm. There were no major differences in apoptotic staining (brown) in infected WT mice (F) and IL-33 KO (G); Bar = 50 μm. (TIF) [file pntd.0004467.s003.tif]

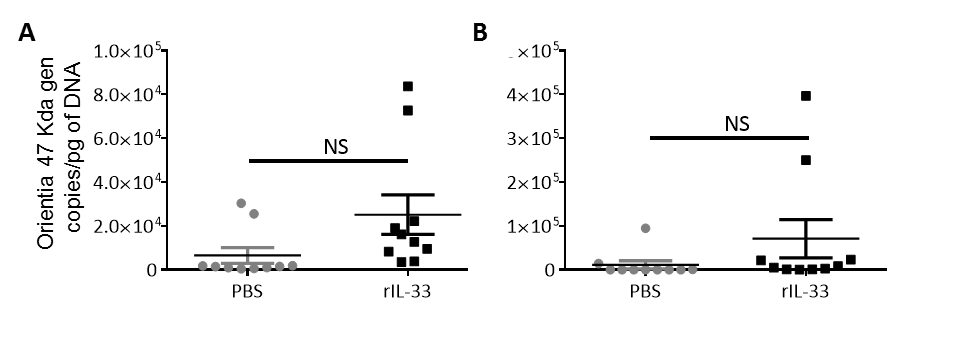

Supplement: S3 Fig — WT mice were infected with sub-lethal dose of O. tsutsugamushi Karp strain and then injected with rIL-33 or PBS every other day, as described in Fig 6. The kidneys (A) and livers (B) were collected from moribund mice (between 10–12 dpi for some rIL-33-treated mice) and terminated mice (13 dpi for the rest mice) for the analysis of tissue bacterial loads by qPCR. NS, no significance. (TIF) [file pntd.0004467.s004.tif]

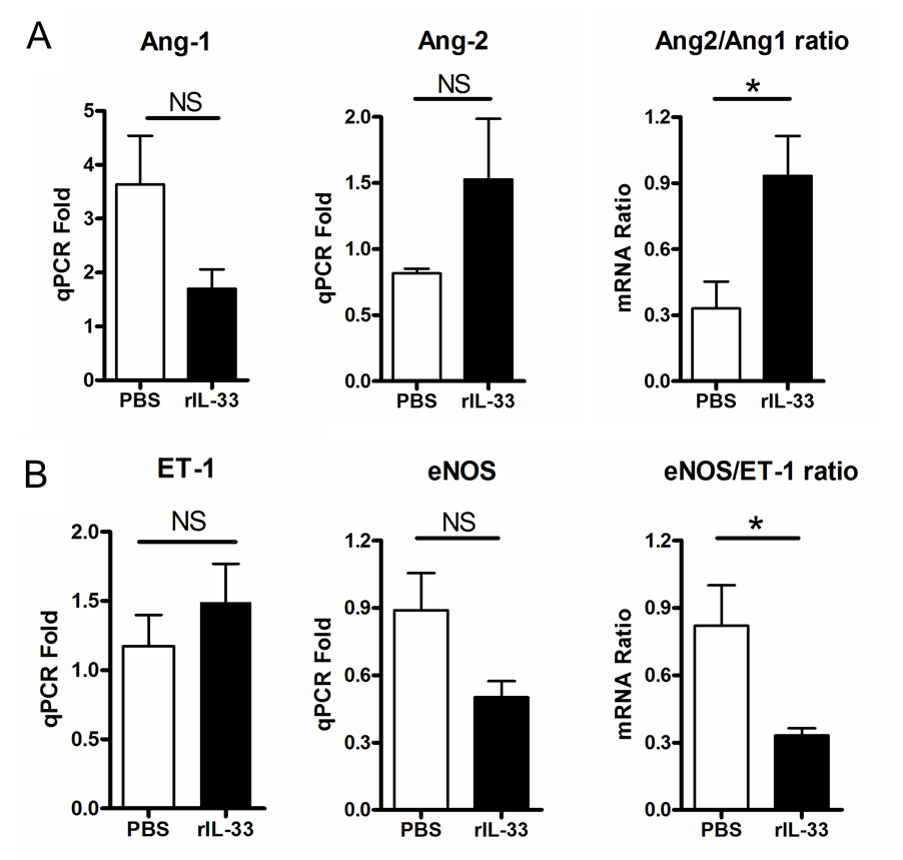

Supplement: S4 Fig — WT mice were infected and treated as in Fig 6. The liver samples were collected at 0 and 9 dpi and analyzed by qRT-PCR for the expression of Ang1, Ang2, and Ang2/1 ratios (A), as well as eNOS, Endothlin-1, and their ratios (B). Data are presented as “qPCR fold” (after normalization to the house-keeping genes), and are shown as mean ± SEM in each group. Representative results are shown from three independent studies with similar trends. *, p < 0.05; NS, no significance. (TIF) [file pntd.0004467.s005.tif]

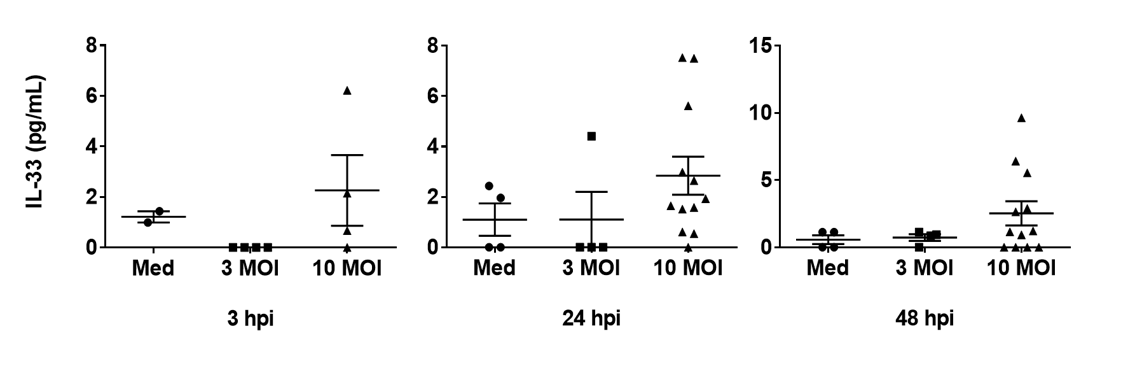

Supplement: S5 Fig — Confluent HUVEC monolayers in 24-well plates were left untreated (Med) or infected with bacteria either at MOI of 3 or MOI of 10, as described in Fig 8. Supernatants were collected from two independent experiments at 3, 24, and 48 hpi and analyzed for IL-33 secretion by using Quantikine ELISA kits. Data are presented as mean ± SEM. There were no significant differences among the infected and control groups. (TIF) [file pntd.0004467.s006.tif]
